# Evaluating the Use of Hydroxychloroquine in Treating Patients With Rheumatoid Arthritis

**DOI:** 10.7759/cureus.19308

**Published:** 2021-11-06

**Authors:** Armaan M Nazir, Bhavya Koganti, Kunal Gupta, Marrium S Memon, Muhammad Bin Aslam Zahid, Vignarth Shantha Kumar, Mamatha Tappiti, Jihan A Mostafa

**Affiliations:** 1 Medicine, California Institute of Behavioral Neurosciences & Psychology, Fairfield, USA; 2 Internal Medicine, California Institute of Behavioral Neurosciences & Psychology, Fairfield, USA; 3 Family Medicine, California Institute of Behavioral Neurosciences & Psychology, Fairfield, USA; 4 Pathology, California Institute of Behavioral Neurosciences & Psychology, Fairfield, USA; 5 Neurosciences, California Institute of Behavioral Neurosciences & Psychology, Fairfield, USA; 6 Psychiatry, California Institute of Behavioral Neurosciences & Psychology, Fairfield, USA

**Keywords:** cardiovascular disease, disease modifying anti-rheumatic drugs, methotrexate, retinopathy, chronic kidney disease (ckd), hydroxychloroquine, rheumatoid arthritis

## Abstract

Rheumatoid Arthritis (RA) is one of the most common autoimmune diseases present today. Although treatment options may differ among clinicians, a commonly prescribed treatment is hydroxychloroquine (HCQ), alone or in combination with other medications. HCQ has been studied for its immunomodulatory effects as well as its role in treating adverse conditions associated with RA. This systematic review examined the use of HCQ therapy in RA patients. A systematic search for relevant literature through PubMed, National Institute of Informatics, Japan (CiNii), and Science Direct databases were carried out in August 2021. Literature directly related to HCQ therapy for RA patients, RA-associated chronic kidney disease, and cardiovascular disease (including lipid profile) was considered relevant. HCQ associated retinopathic adverse effects were also selected for this review. Thirty-eight articles were found to be relevant, passed quality assessment, and were included in this review. Nine articles discussed HCQ therapy in comparison with other therapies (mainly methotrexate and sulfasalazine), but were contradictory in their outcomes, as were the seven papers that reviewed kidney function in RA patients with and without HCQ. Five articles credited better cardiovascular outcomes to RA patients taking HCQ. Sixteen articles studied the relationship between HCQ and retinal toxicity, providing insights into the risks associated with HCQ therapy. HCQ therapy was found not only to be beneficial in slowing the disease progression in RA patients but enhanced the effects of methotrexate in treating RA as well. Data strongly associates HCQ therapy with the mitigation of RA-related cardiovascular and kidney conditions. However, if HCQ is prescribed, it is imperative to be aware of the possible (although rare) retinopathic adverse effects associated with this therapy.

## Introduction and background

Rheumatoid Arthritis (RA) is the most prevalent autoimmune inflammatory disease present today, primarily affecting people between 20-50 years of age, although children as well as the elderly may also be susceptible [1]. It is a chronic form of arthritis, affecting joints in the body bilaterally, and has been estimated to effect over 1.3 million people in the United States alone [1]. The reason behind RA onset is multifaceted, with genetics (human leukocyte antigen-DR Beta 1 (*HLA-DRB1*) gene), environmental conditions (smoking, alcohol, birthweight, socio-economic regions of birth), as well as hormones (primarily estrogens) playing an important role [2-4].

Although RA primarily affects the joints, it is known to frequently affect extra-articular structures including, but not limited to, the heart, vascular structures, and kidneys [5,6]. As RA is incurable, its management is of critical importance, and along with this comes the need to manage all extra-articular associated conditions as well.

RA is conventionally treated with a group of drugs known as disease-modifying anti-rheumatic drugs (DMARDs), which include methotrexate (MXT), sulfasalazine (SSZ), and hydroxychloroquine (HCQ) [7-10]. However, the question of the best treatment option for RA patients still remains unanswered. Common interventions include MTX or HCQ monotherapy, MTX-HCQ or MTX-SSZ dual therapy, and MTX-HCQ-SSZ triple therapy, although other variations of these DMARDs as well as less common DMARDs are also prescribed [7-10].

Interestingly, HCQ, an analog of chloroquine was initially marketed as an antimalarial drug used to treat malaria but has since been replaced by more effective drugs. However, it was later identified as a DMARD in its role as an immune system modulator and has been since used to treat autoimmune diseases including systemic lupus erythematosus and RA. HCQ has various immunomodulatory effects, which include the inhibition of phagocytosis and chemotaxis, toll-like receptor signaling, calcium signaling in lymphocytes, macrophage-mediated cytokine production, and matrix metalloproteinases [11]. It has been found not only to help in treating RA, but also has the potential to help mitigate its associated extra-articular conditions.

Common scales to measure improvement and deterioration exist for patients suffering from RA, The Rheumatoid Arthritis Disease Activity Index (RADAI), supplemented by the disease activity score (DAS28), measures four aspects; 28 tender joint counts, 28 swollen joint counts, erythrocyte sedimentation rate (ESR), and general patient health [12]. The (American College of Rheumatology/ European League Against Rheumatism (ACR/EULAR) criteria measures four aspects as well, namely; joint involvement, serology test results, acute phase reactant test results, and patient reporting of RA signs and symptoms (self-reporting), and is used to assess even early arthritis [13]. This evaluation allows for quick treatment before the RA disease progresses too far [13].

In the current scheme of RA treatment, there is a lack of clarity as the medical community is disputed in its attempt to establish an optimum treatment plan for RA patients. Previous literature has highlighted the use of HCQ in combination or comparison with other DMARDs (MXT in particular), as well as its ability to treat the conditions associated with RA separately, in an attempt to ascertain the effectiveness of HCQ as a DMARD. Other literature has also highlighted the retinopathic risks associated with HCQ use. However, to the best of our knowledge, no systematic review has attempted to combine these factors to provide a complete analysis of HCQ as a treatment option for RA patients. With multiple treatment options now available for RA, a detailed integrated understanding of these various aspects was lacking and is required to fully understand the potential for HCQ in RA therapy, so that medical practitioners can make an informed decision when considering HCQ as a treatment option.

## Review

Methods

In August 2021, literature relevant to this topic was systematically searched through three databases; PubMed, Science Direct, and CiNii. In PubMed, a specific search strategy was developed through the Medical Subject Headings (MeSH) keywords. The basic MeSH keywords used were "therapeutic," "arthritis," and "hydroxychloroquine." However, an extensive MeSH keyword search was generated to streamline the results (Table 1). 

**Table 1:** Generating MeSH keywords MeSH: Medical Subject Headings

Keywords	MeSH Keyword
Therapeutic	( "Therapeutics/adverse effects"[Majr] OR "Therapeutics/drug effects"[Majr] OR "Therapeutics/drug therapy"[Majr] OR "Therapeutics/therapeutic use"[Majr] ) OR Treatment
Arthritis	("Arthritis, Rheumatoid/analysis"[Majr] OR "Arthritis, Rheumatoid/anatomy and histology"[Majr] OR "Arthritis, Rheumatoid/complications"[Majr] OR "Arthritis, Rheumatoid/drug therapy"[Majr] OR "Arthritis, Rheumatoid/prevention and control"[Majr] OR "Arthritis, Rheumatoid/rehabilitation"[Majr] OR "Arthritis, Rheumatoid/therapy"[Majr])
Hydroxychloroquine	("Hydroxychloroquine/administration and dosage"[Majr] OR "Hydroxychloroquine/adverse effects"[Majr] OR "Hydroxychloroquine/analogs and derivatives"[Majr] OR "Hydroxychloroquine/therapeutic use"[Majr]) OR Hydroxychloroquine
Final PubMed Search	1 AND 2 AND 3

The remaining databases were searched using basic keywords including "effectiveness," "treatment," "hydroxychloroquine," and "rheumatoid arthritis." The results were first filtered for initial inclusion by text availability, article type, publication date, species and language (Table 2). Of the included literature, the titles or abstracts directly related to the research question were further selected for a full text review. 

**Table 2:** Initial inclusion criteria

Criteria	Selected Criteria for Inclusion	Justification
Text Availability	Full Text Availability	Literature with partial or incomplete texts were excluded as considered an inadequate representation of the literature.
Article Type	Observational Studies	To allow for easy interpretation of this systematic review paper, only observational studies, having similar modes of data collection and analysis were selected. All forms of observational studies were used.
Publication Date	2016-2021	Articles published before the chosen date range were excluded as they were considered inaccurate representations of current day therapeutics as compared to papers published in the last five years.
Language	English	All investigators were fluent in English only.
Species	Humans	This review studies the use of hydroxychloroquine in treating rheumatoid arthritis in humans only.

Information directly concerning the use of HCQ as a treatment for RA was considered relevant. Literature focused on the adverse effects of HCQ were also considered relevant. Literature regarding HCQ alone or any other form of arthritis besides RA were excluded from the search. Studies focused on interventions besides HCQ, but merely referring to HCQ as an alternate intervention were also excluded from the search. This was left to the investigator’s discretion. Age, although a factor in the development of RA was not considered as an inclusion/exclusion criteria [1], as it bore no relevance to the research question, which focused on the treatment of RA with HCQ, rather than the disease itself.

The structure of the search strategy was developed in strict adherence to the Preferred Reporting Items for Systematic Reviews and Meta-Analyses (PRISMA) 2020 checklist. The quality of the chosen full-text literature was assessed using a two-step strategy. All of the selected literature was first screened through the Newman-Ottawa Scale, with a focus on case definition, assessment of outcome (including analysis of results), as well as adequacy of follow-up of cohorts, where follow-up time deemed inadequate to produce reliable results was excluded. This too was left to the investigator’s discretion. Selection through the Newman-Ottawa Scale was carried out independently by two investigators to reduce bias, and only literature deemed to be of high quality as per the Newman-Ottawa Scale by both investigators was selected. Qualitative observational studies were further appraised by the Critical Appraisal Skills Programme (CASP) tool while observational cohort and cross-sectional studies were appraised through the Study Qualitative Assessment Tools developed by the National Heart, Lung, and Blood Institute (NHLBI). Articles that met all the above criteria were used in this Systematic Review.

Results

The preliminary search produced 189 results from PubMed, 66 results from Science Direct and nine results from CiNii, giving a total of 264 results that met the criteria for initial Inclusion (Table 2). The full-text of one article from PubMed and one article from CiNii (in Japanese) could not be recovered, and so were excluded. One article was duplicate, found through both PubMed and Science Direct searches, and the duplicate was removed [14]. Of the remaining 262 articles, 72 abstracts (62 from PubMed, seven from Science Direct and three from CiNii) were selected for a full-text review and assessed for relevance. Of these, 20 articles were considered relevant and their references were reviewed for additional relevant literature not found through the initial database searches. In the review of the references of the finalized 20 articles, an additional 26 articles were considered relevant to the research question and included in this paper. Additional studies relevant to the research question, presented as evidence within the selected observational studies were selected regardless of the type of study, as they are used in this systematic review only to support data from the selected observational studies.

**Figure 1:**
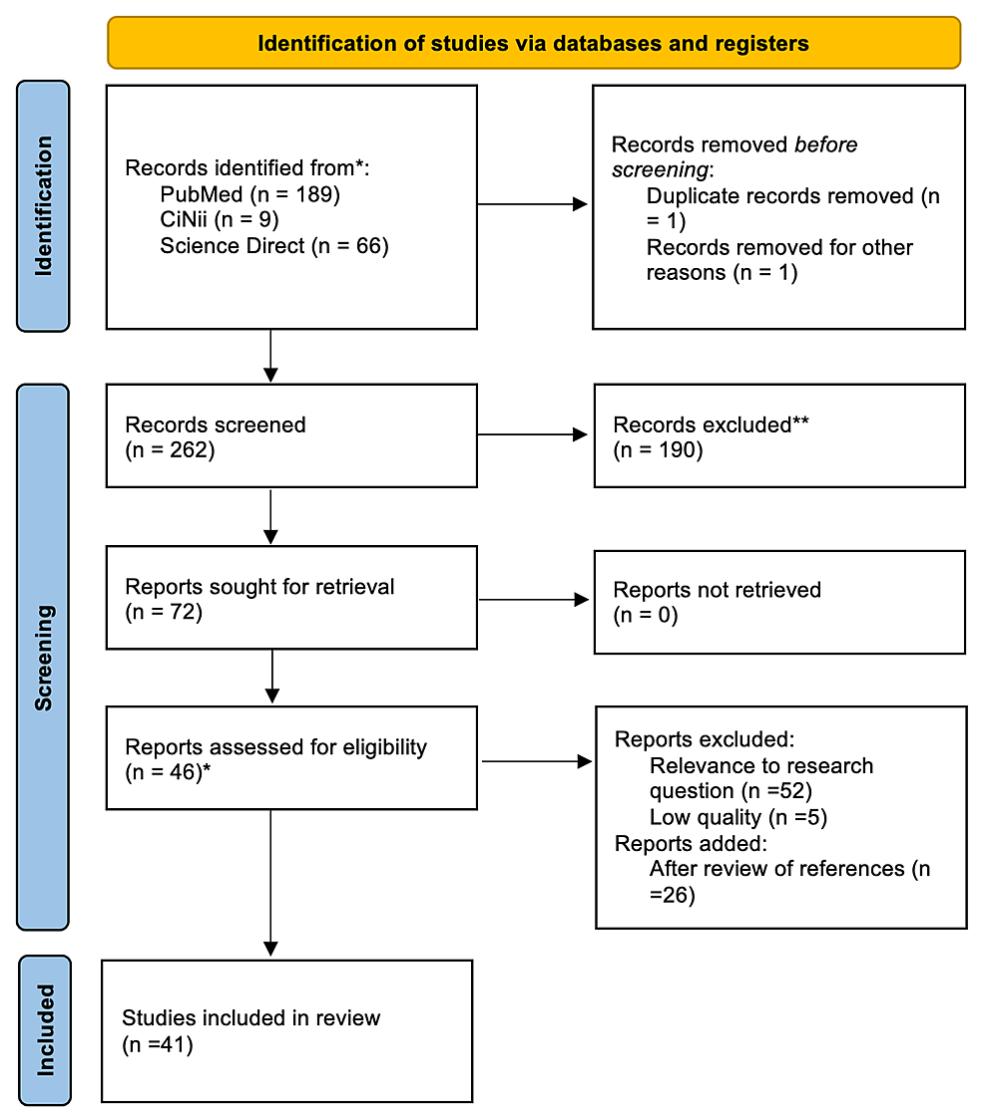
Prisma flow diagram N = Number Adapted from: Swartz (2020) [20]

The combination of both selected observational studies as well as additional articles were assessed for quality (20 observational studies + 26 additional studies = 46 studies in total). Five papers were considered low quality and excluded from this paper [15-19]. A total of 41 studies was finally selected for this systematic review (Figure 1).

Of the 41 articles, eight articles discussed HCQ therapy in comparison with other therapies (mainly MTX and SSZ); 11 articles studied the relationship between HCQ, RA, and cardiovascular disease (CVD), of which five articles credited better cardiovascular outcomes to RA patients taking HCQ; six papers that reviewed kidney function in RA patients with and without HCQ; and 16 articles studied the relationship between HCQ and retinal toxicity (Table 3).

**Table 3:** Distribution of included literature HCQ:. hydroxychloroquine; CVD: cardiovascular disease; CKD: chronic kidney disease

Subheadings	No of Articles N= 41	Article References
HCQ Therapy	8/41= 19.5%	7-10, 22-25
CVD	11/41= 26.8%	26-31, 34-36
CKD	6/41= 14.6%	5, 39-43
Retinopathy	16/41= 39%	44-59

Discussion

Hydroxychloroquine Therapy

A study conducted by Bergstra et al. on 5535 patients with RA (women n= 4393, men n=1142) found through the Measurement Efficacy of Treatment in the ‘Era of Outcome’ in Rheumatology (METEOR) database that while women were on average 12.6% more likely to begin initial treatment with HCQ monotherapy or in combination with either MXT or glucocorticoids, men were more likely to be prescribed MXT alone, or in combination without HCQ [6]. Although differences in patient response and outcome to similar treatments was found to be insignificant between both sexes, the disparity in HCQ prescription as an initial treatment for RA suggests an ambiguity amongst the medical community in its role as an effective treatment against RA.

In a controlled quasi-experimental study, Schapink et al. investigated the value of HCQ-MXT combined therapy versus MXT monotherapy in 325 early RA patients [8]. After six months, a DAS28-CRP difference of 0.38 points (CI: 0.010, 0.76) was observed in favor of patients in the MTX-HCQ combination therapy group, as well as a 15% difference in the number of patients with a good EULAR score (61% MTX-HCQ vs 46% MTX), both showing significantly better outcomes for patients receiving HCQ in addition to standard MTX in the short term (less or equal to six months). However, at the 12-month mark, the ∆DAS28-CRP (-0.22; CI: -0.19, 0.62) was no longer significant, and neither was the difference in good EULAR responses (∆= 6%). As established by Carmichael et al., HCQ supports a greater exposure to MTX, which may justify the improvement seen in the MTX-HCQ cohort in the short term only [21].

This hypothesis is further supported by the work of Klarenbeek et al., where MTX-SSZ combination therapy showed no better outcome as compared to MTX monotherapy, yet the MTX-SSZ-HCQ combination therapy showed better performance than MTX monotherapy [10]. This study further establishes HCQ as the defining therapeutic responsible for improved good EULAR responses and overall improved outcomes for RA patients, at least in the short term (less or equal to six months).

The above data seems to suggest a negligible improvement, if at all, with the addition of HCQ in treating RA in the long run [8]. Regardless, the importance of treatment in the initial stages of early RA, or a “therapeutic window of opportunity early in the disease course” is an established critical factor in controlling the disease progression [22-24]. Hence, although it may seem ultimately futile to prescribe HCQ in efforts to see long term improvements in RA patients, this is actually not the case, and it would be beneficial to avail of the short term benefits of adding HCQ to the initial treatment plan, as an investment towards diminishing the course of the RA disease progression in the long term.

It may be worthwhile to note that the study conducted by Wabe et al. took a look at the disease activity trajectories in early RA patients, associated with persistence to treatment [9]. In this three year study, 297 patients were observed and assessed at the 12, 18, 24, 30, and 36 month marks, and it was found that while strict adherence to treatment was met with decreased disease activity trajectories for MTX, HCQ, as well as SSZ, combined therapy of MTX-HCQ saw the highest percentage (80%) persistence (adherence to treatment) throughout all five follow-ups [9]. Consequently, it would appear that HCQ, if added to the RA treatment plan, would not be detrimental to the patient’s adherence to the treatment, effectively mitigating any fears over increased failure in adherence to treatments that include HCQ.

Cardiovascular Disease and Lipid Profile

In RA patients, the most common cause of death is from CVD [26]. In addition, CVD is seen in greater numbers in RA patients than in non-RA patients [26-30]. Hyperlipidemia has been established as one of the most important and modifiable risk factors in the development of CVD. This being said, lipid profile control can be an invaluable influencer in mortality control with RA patients [31,32]. Five papers analyzed the association between cardiovascular events and HCQ use in RA patients [33-37]. Of these five, all found an improvement in either lipid profile or cardiovascular events seen in RA patients taking HCQ regularly. This was not seen for any other DMARD.

**Figure 2:**
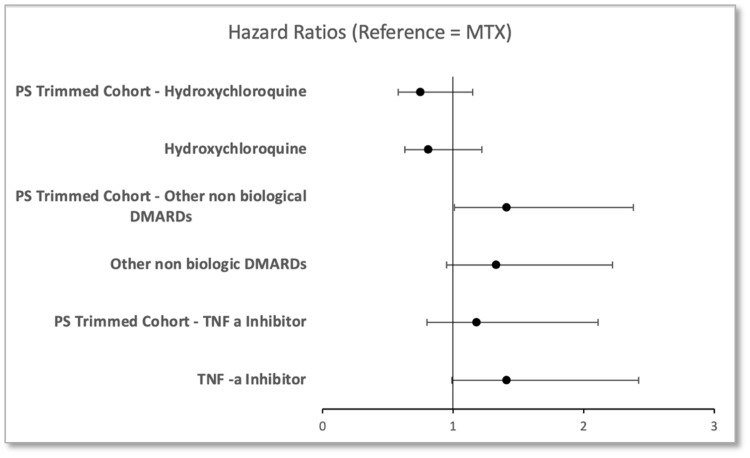
Hazard ratios – forest plot (MTX as reference (HR=1)) MTX: methotrexate; PS: propensity score; DMARD: disease-modifying anti-rheumatic drugs; TNF-α: tumor necrosis factor alpha; HR: hazard ratio Source: Desai et al. (2015) [33].

In a retrospective cohort study conducted by Desai et al., 17,145 RA patients were assessed for hyperlipidemia, of which 35.75% took HCQ, 46.32% took MTX, and 17.93% various others (TNF- α etc.) [33]. The incident rate of hyperlipidemia was found to be lowest in HCQ users (20.1 cases per 1000 person-years; (95% CI, 16.3-24.6)), with HR 0.81 (95% CI, 0.63-1.04) compared to MTX (Figure 2). 

The propensity score (PS) trimmed cohort further confirmed these results, with HR 0.75 (95% CI, 0.58-0.98) for HCQ in comparison to MTX (Figure 2) [33]. Other non-biologic DMARDs saw an increased incidence of hyperlipidemia as compared to MTX (HR= 1.41; 95% CI, 1.01-1.98) and therefore HCQ as well. HCQ use was also associated with statistically significant reductions in low-density lipids (LDLs), cholesterol, as well as triglycerides, compared to MTX, observed in three papers supporting previous literature [31-35]. It is clear that HCQ therapy provides a significant advantage in dealing with modifiable cardiovascular risk factors associated with RA. An average HR of 0.78 (of raw and PS trimmed cohorts) proves the distinctive advantage HCQ treatment provides in improving risk factors, namely: lipid profile (reduced hyperlipidemia), reduced LDL, cholesterol, and triglycerides; all major contributing factors toward CVD [31-35].

A large retrospective cohort study conducted by Sharma et al. studying 1266 RA patients (547 HCQ users and 719 HCQ nonusers) established a striking 72% decrease in the incidence of CVD in patients taking HCQ ( HR= 0.28; 95% CI, 0.12-0.60; p= 0.002) and 70% decrease in the incidence of composite coronary artery disease, stroke, and transient ischemic attack as compared to HCQ nonusers [36]. This 12-year study is clear in its findings, showing substantial benefits for RA patients using HCQ in dealing with the cardiovascular complications associated with RA. Similar results were obtained from the cross-sectional cohort study conducted by Li et al., where RA patients with CVD incidence was seen in the smallest percentage in patients taking HCQ (9.4%) compared to patients taking MTX (37%) [37].

This being said, the above literature when interpreted together provides a strong argument in favor of medical practitioners prescribing HCQ not in its role as a treatment against RA directly, but as an important influencing factor in preventing often fatal cardiovascular complications commonly associated with RA [31-33,36,37].

Chronic Kidney Disease

The relationship between RA and chronic kidney disease (CKD) has previously been cited in a lot of literature [5,38,49]. The possibility of reduced kidney function is now well established in the disease progression of RA, with complications such as mesangial proliferative glomerulonephritis seen in 34-36% of patients with RA, often a precursor to CKD [40,41]. In addition, studies such as the retrospective study on 118 individuals conducted by Landewé et al. showed a 10% reduced creatinine clearance rate after the use of HCQ, suggesting the nephron-toxic nature of HCQ [42].

However, a recent larger observational cohort study by Wu et al. consisting of a total 2619 patients (1212 hydroxychloroquine users) showed a significantly lower (36%) incidence of CDK in patients treated with HCQ than HCQ non-users (HR 0.64; 95% CI, 0.45 to 0.90; p= 0.01) [5]. This was seen in the short term as well as the long term. Furthermore, an inverse dose-response relationship was found between HCQ use and the incidence of CKD, with >70 cumulative defined daily dose showing a 40% lower propensity for developing CDK (HR - 0.37; 95% CI, 0.16-0.84; p= 0.02) than less or equal to 35 cumulative defined daily dose (HR - 0.77; 95% CI, 0.47 to 1.21; p= 0.25) [5]. With the protection of kidney function a growing concern among patients with RA, the study conducted by Landewé et al. paints a bleak picture of HCQ use as a drug intervention for RA patients [42]. However, the study by Wu et al. clearly contradicts the study conducted by Landewé et al., which is limited by its small sample size (118 patients vs 2,619) and rather suggests the beneficial outcomes that can be expected with HCQ use with respect to kidney function [5,42].

HCQ Associated Retinopathy

HCQ use over a long period of time has been linked with retinopathy in the literature [43-46]. Theories for this include HCQ binding onto melanin within the renal-pigmented epithelium (RPE), as well as the inhibition of all trans-retinol uptake resulting in negative changes in the visual cycle [44,47]. Even more discouraging is that HCQ related retinal toxicity may also be irreversible, with continued retinopathy seen even after the suspension of the drug [45,48,49]. In agreement with Schroeder et al. [44], a retrospective cohort study conducted by Lee et al. [50] saw that while retinopathy progressed while on HCQ, the progression stopped in patients with toxicity detected prior to RPE damage.

A study conducted by Yen et al. saw a higher OR for developing retinal disorder in patients treated with HCQ (OR= 1.67; 95% CI, 1.20-2.30) [51]. The OR (1.67) was significant, implying an increased probability of developing retinal toxicity while taking HCQ. Likewise, Tarakcioglu et al. studied 53 patients with RA and 32 patients in the control group for signs of retinopathy over a period greater than five years, as part of a cross-sectional cohort study [52]. It was found that while all vascular density values were similar between both groups, superficial and deep whole thicknesses, para-foveal thicknesses as well as peri-foveal thicknesses were lower in patients treated with HCQ, indicating the onset of retinopathy [52]. The majority of these numbers were seen in patients taking HCQ for greater than five years. 

These results are further confirmed in studies conducted by Ulviye et al. and Eo et al., characterizing the onset of retinal toxicity by HCQ with reduced inner and outer retinal thicknesses as an identifiable indicator [53,54]. However, the work of Melles et al. concluded that ethnicity played a hand in the pattern of retinopathy development, whether peripheral (seen frequently in Asian ethnicities) or otherwise [55]. Hence, taking ethnicity into consideration, conclusions drawn by Tarakcioglu et al., Ulviye et al. and Eo et al. regarding the peri-central (peripheral) nature of HCQ related retinal toxicity cannot be considered reliable against any ethnic population besides that studied in each individual paper [52-54]. Yet multiple papers with similar results still point towards peri-central retinopathy being an identifiable characteristic of HCQ induced retinal toxicity, and it is important for medical practitioners to be aware of this.

While previous studies maintain that retinopathy is very rare, studies conducted by Wolfe et al., Lee et al., and Melles et al. saw retinal toxicity prevalence as high as 0.65%, 4.1%, and 7.5% respectively in patients treated with HCQ in the long term ( ≥three years) [56,50,57]. The lack of homogeneity among these results questions their reliability and warrants further investigation, preferably using larger sample sizes to provide accurate reproducible data that can be used to definitively outline the risks while using HCQ to treat RA.

Nika et al. found that out of 1409 patients using HCQ for a time period of more than four years, 27.9% lacked routine eye check-ups, while 34.5% had had no diagnostic testing for maculopathy in the entire five year period [58]. With a moderately low risk of developing retinopathy and accurate ophthalmologic testing available, the benefits of HCQ far outweigh the risks. Yet, seeing that the risk of retinopathy is well established, a severe prognosis worsened by its permanence after cessation of HCQ, it is imperative that medical practitioners prescribing HCQ insist on regular ophthalmology screening tests for retinopathy, regardless of the HCQ treatment duration.

Limitations and future studies

There were several limitations in this study. Only observational studies were used as primary literature to allow for easy integration and interpretation of data. However, it is possible that clinical trials may invaluably help in quantifying the differences between different therapies. This may include different HCQ doses, or in comparison with MTX doses. Clinical trials could additionally provide better understanding of the pharmacokinetics and pharmacodynamics of HCQ within the body, all of which we were unable to include in this systematic review, which may help fine-tune our understanding of HCQ dosing and prescription.

Although two investigators were used to minimize bias, quality assessment was left to the investigators' discretion and the elimination of all bias was impossible. Furthermore, many papers were contradictory in their results, and although a reason for this can be speculated, without more reproducible data it is justified to assume that conclusions drawn are neither undeniably accurate nor free from personal bias.

For example, the effect of HCQ on kidney function has been poorly studied. Although literature exists, the articles found were in clear contradiction of each other and, without sufficient support from supplemental literature, a compelling conclusion could not be made. Thus, the inadequacy of sufficient data regarding HCQ and kidney function in RA patients proved to be a significant limiting factor, rendering us unable to adequately determine the role HCQ would play in this regard. There is a desperate need for further investigation into this area, owing to the paucity of available information.

The distribution of included literature among the subheadings was also uneven with ≈40% of selected articles used in the analysis of HCQ retinopathy, and only 14.6% of all articles used to discuss HCQ and CKD (Table 3). This causes unevenness in the strengths of analyses allowing for evidence and conclusions under one subheading to be superior to another. This disparity could serve as a limitation in that readers may find it harder to gauge the reliability of the individual analyses with respect to each other.

Some papers mentioned a difference between HCQ use in the long versus short term [8]. However, this wasn’t extensively analyzed as part of this systematic review but could provide further insights into HCQs use, and possibly find an optimum duration for HCQ treatment. In the future, if HCQ duration is studied with a focus on the aspects of HCQ and RA studied in this systematic review (CVD, CKD, comparative effectiveness, and HCQ-related retinopathy), the results would only improve clinicians’ abilities to provide accurate HCQ therapy to their patients.

## Conclusions

RA is widely prevalent across the globe, yet no optimum treatment plan exists. This systematic review aimed to evaluate the use of HCQ in treating RA. The paper focused not only on the ability of HCQ to treat the primary articular-related disease progression, but also its associated symptoms.

Studies suggest the use of HCQ alone or in combination with other DMARDs in order to decrease initial disease progression, keeping in mind that strong treatment at the initial stage of RA is a critical "window of opportunity" to control the disease. This paper found a dispute in the role HCQ plays in CKD. However, stronger evidence suggested a 36% lower incidence of CKD seen in RA patients taking HCQ. CVD is one of the most common complications and the leading cause of death in RA patients. While comparing treatment options, it was clear that HCQ provided significantly greater protection against CVD, to a much higher degree than any other DMARD. Although rare, retinopathy is a severe adverse effect that affects some patients taking HCQ, hence it is strongly recommended that HCQ users undergo regular ophthalmologic check-ups to ensure no retinal toxicity.

To the best of our knowledge, this paper is the first to provide a complete evaluation of the use of HCQ in treating RA. Not only did it detail the ability of HCQ to improve primary RA treatment, but it also analyzed the role of HCQ in treating the common conditions associated with RA, without losing sight of its risks. Although separate analyses regarding HCQ treatment of certain aspects of RA disease progression exist, RA is a multi-pronged disease and in order to comprehensively appraise the use of HCQ in treating RA patients, it was important that all discussed aspects of HCQ use were integrated together so clinicians can make informed decisions when considering HCQ therapy, which this systematic review has provided.
